# Abscopal Activation of Microglia in Embryonic Fish Brain Following Targeted Irradiation with Heavy-Ion Microbeam

**DOI:** 10.3390/ijms18071428

**Published:** 2017-07-04

**Authors:** Takako Yasuda, Miyuki Kamahori, Kento Nagata, Tomomi Watanabe-Asaka, Michiyo Suzuki, Tomoo Funayama, Hiroshi Mitani, Shoji Oda

**Affiliations:** 1 Department of Integrated Biosciences, Graduated School of Frontier Sciences, The University of Tokyo, Kashiwa, 277-8562 Chiba, Japan; 8656689177@edu.k.u-tokyo.ac.jp (M.K.); 9510279742@edu.k.u-tokyo.ac.jp (K.N.); twatana@edu.k.u-tokyo.ac.jp (T.W.-A.); mitani@k.u-tokyo.ac.jp (H.M.); odasho@edu.k.u-tokyo.ac.jp (S.O.); 2 Takasaki Advanced Radiation Research Institute, Quantum Beam Science Research Directorate, National Institutes for Quantum and Radiological Science and Technology, 370-1292 Gunma, Japan; suzuki.michiyo@qst.go.jp (M.S.); funayama.tomo@qst.go.jp (T.F.)

**Keywords:** abscopal effects, microglia, phagocytosis, l-plastin, apolipoprotein E, *in situ* hybridization, apoptosis, medaka, ionizing irradiation

## Abstract

Microglia remove apoptotic cells by phagocytosis when the central nervous system is injured in vertebrates. Ionizing irradiation (IR) induces apoptosis and microglial activation in embryonic midbrain of medaka (*Oryzias latipes*), where apolipoprotein E (ApoE) is upregulated in the later phase of activation of microglia In this study, we found that another microglial marker, l-plastin (lymphocyte cytosolic protein 1), was upregulated at the initial phase of the IR-induced phagocytosis when activated microglia changed their morphology and increased motility to migrate. We further conducted targeted irradiation to the embryonic midbrain using a collimated microbeam of carbon ions (250 μm diameter) and found that the l-plastin upregulation was induced only in the microglia located in the irradiated area. Then, the activated microglia might migrate outside of the irradiated area and spread through over the embryonic brain, expressing ApoE and with activated morphology, for longer than 3 days after the irradiation. These findings suggest that l-plastin and ApoE can be the biomarkers of the activated microglia in the initial and later phase, respectively, in the medaka embryonic brain and that the abscopal and persisted activation of microglia by IR irradiation could be a cause of the abscopal and/or adverse effects following irradiation.

## 1. Introduction

Microglia are resident immune cells in the central nervous system (CNS) of vertebrates. They are highly specialized phagocytic cells that act to defend the CNS and are mainly responsible for the clearance of endogenous cell debris in the CNS [1,2,3,4,5]. Microglia also have a very wide variety of physiological functions in neurogenesis and are prerequisite for the normal development and maintenance of the structure and function of the CNS [4,6,7]. The abnormality of microglial functions causes multiple disorders in neural functions and neuropsychiatric diseases [3].

Zebrafish (*Danio rerio*) has been intensively used as a model system for vertebrate during the last decades and its larvae have been an excellent model system for the study of microglia [8]. Since zebrafish larvae are small and highly transparent, and it is easy to establish transgenic fish with fluorescently tagged microglia [9,10], a large number of studies imaging microglia *in vivo* have been conducted in the brain of living larvae to reveal the dynamics of microglia. Imaging of microglia *in vivo* also revealed that microglia are highly dynamic, even in the resting state: microglia are continuously patrolling the surrounding environment with highly motile processes in the mouse brain [11,12] and the dynamic nature of resting microglia was subsequently confirmed also in zebrafish [13]. When the brain is injured or damaged, microglia respond to any kind of brain injury or damage and accumulate at the lesion and start to proliferate [14,15,16,17]. They migrate toward dying neurons or invading pathogens or tumor cells [18,19,20]. Nucleotides released by apoptotic cells, such as ATP, have been identified as a “find-me” signal for microglia [11,21,22]. While the shape of resting microglia is ramified, activated microglia change their morphology to amoeboid and increase their motility [23,24,25,26]. The precise process and molecular machinery of engulfment of dying neurons are clarified in the living larval brain of zebrafish [27,28,29].

Microglia play a Janus-faced role in the damaged CNS. While their primary role is the clearance of apoptotic cell debris as described above, activated microglia also release cytokines and cause inflammation in the CNS. When the microglia-induced neuroinflammation persists, it affects CNS functions. Cranial radiation therapy (CRT) is a widely-accepted treatment for intracranial tumors, because it is highly noninvasive compared with surgical resection. However, CRT has a significant possibility to induce radiation-induced brain injury (RIBI) in the healthy tissues surrounding the tumor. Clinical studies demonstrated that acute neural detrimental effects such as cognitive impairment can be induced in patients after CRT [30,31,32] and neuroinflammation may be caused by microglial activation following irradiation [33,34,35].

Besides the local effects of CRT at the cellular and molecular levels in tumor tissues, the effects of CRT on the host’s immune system are nowadays at the center of interest of both clinical and basic investigators. These effects can be strong enough to immunize the patient against the tumor, leading to a rejection of both the irradiated tumor and distant metastases by the host, the so called “abscopal effect” [36,37,38]. Recently, increasing attention has been paid to the abscopal effects of cancer killing, and to the application of CRT in combination with various immunotherapies [39,40,41]. To minimize the adverse effects on normal tissue surrounding the tumor during CRT, understanding and regulation of irradiated microglial dynamics would be highly advantageous; however, these issues have yet to be fully addressed.

Medaka fish is a popular model of humans in life science [42]. It is small (3 cm adult body length) and whole-body histological sections enable us to examine it histopathologically [43,44]. Like zebrafish larvae, medaka larvae are small and highly transparent, so apoptotic neural death can be clearly visualized throughout the whole brain [45,46,47,48]. This small fish has been an important vertebrate model with which to study the effects of acute ionizing irradiation (IR) on gonadal and various somatic tissues including neuronal cells [44,46,49,50]. We have shown that microglia in the irradiated larval brain of medaka are principally responsible for the clearance of apoptotic cell debris via their phagocytotic activity [5].

Here, using medaka embryos as a vertebrate model system, we established experimental procedures to irradiate the embryonic optic tectum (OT) locally, using a collimated carbon-ion beam system at the facility of Takasaki Ion Accelerators for Advanced Radiation Application (TIARA) in National Institutes of Quantum Beam Science and Technology (QST), and investigated the dynamics of microglial activities induced after the irradiation. We demonstrated that the microglial activation includes two steps throughout the phagocytotic process, which can be indicated by the specific expression of l-plastin (lymphocyte cytosolic protein 1) and apolipoprotein E (ApoE). Furthermore, microglial activation was induced, not only in the target irradiation area of OT, but also in the nonirradiated area of OT. These findings strongly suggest that the abscopal activation of brain immune system can be induced after CRT and that the medaka embryonic brain is a promising model system with which to investigate the dynamics of microglia activities in the irradiated embryonic brain.

## 2. Results

### 2.1. Sequential Process of Microglial Activation Following γ-Ray Irradiation with 10 Gy

**Figure 1:**
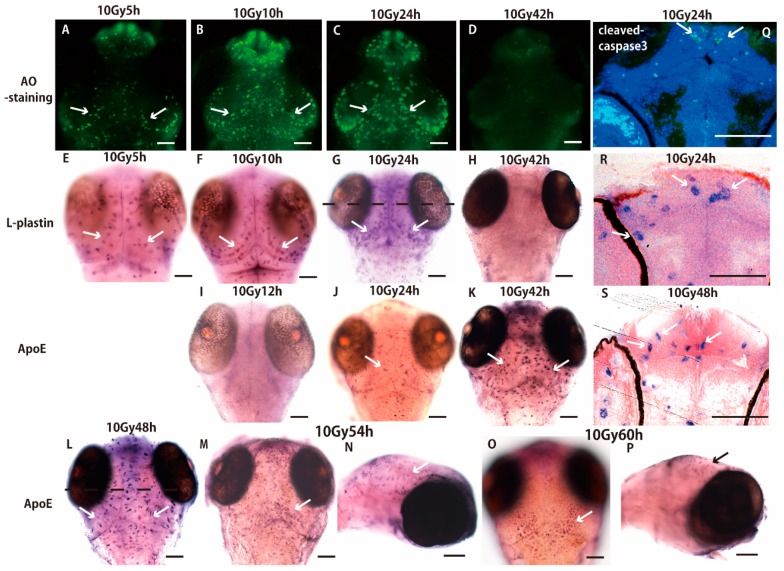
Sequential process of microglial activation following γ-ray irradiation. Acridine orange (AO)-positive apoptotic neurons started to form rosette-shaped clusters 5 h after the irradiation of γ ray (10 Gy) (**A**). Number of clusters increased and they were located in the marginal area of the optic tectum (OT) 10–24 h after the irradiation (**B**,**C**) then disappeared 42 h after the irradiation (**D**). The distribution of l-plastin mRNA revealed by Whole-Mount *In Situ* Hybridization (WISH) was identical to that of AO-positive apoptotic neurons during 5–42 h after the irradiation (**E**–**H**). In a histological section of the WISH-processed brain 24 h after the irradiation (**G**) prepared at the dotted line, l-plastin expressing microglia were distributed in the marginal area of the irradiated OT (arrows in **R**) in the same manner as that of cleaved-caspase 3 positive apoptotic neurons (arrows in **Q**). By contrast, no ApolipoproteinE (ApoE)-expressing microglia were present 12 h after the irradiation (**I**). ApoE-expressing microglia started to appear 24 h after the irradiation (arrow in **J**) and they increased throughout the whole brain 42–48 h after the irradiation (arrows in **K**,**L**). A histological section of the WISH-processed brain 48 h after irradiation (**L**) prepared at the dotted line in **L** shows that the activated microglia changed their cell appearance from ramified to amoeboid morphology (arrows in **S**). Number of ApoE-expressing microglia decreased 54 h after the irradiation (arrows in **M**,**N**) and completely disappeared within the brain, but accumulated on the dorsal surface of the brain 60 h after the irradiation (arrow in **O**,**P**). AO-stained and WISH-processed brains in **A**–**M** and **O** show dorsal views, and (**N**,**P**) show lateral views. Scale bars = 100 μm.

We investigated the sequential process of microglial activation induced by γ-ray irradiation of the brains of medaka embryos (stage 28, 3 days after fertilization), focusing on the time course of maintenance and termination of the microglial activation by whole-mount *in situ* hybridization (WISH) with antisense RNA probes of l-plastin and ApoE. Acridine orange (AO)-positive apoptotic neurons, which were visualized as scattered AO-positive spots over the entire area of the OT in the irradiated embryos 3 h after the irradiation, started to form the rosette-shaped clusters 5 h after the irradiation (Figure 1A). These clusters increased in number, enlarged, and were located in the marginal area of the OT 10–24 h after the irradiation (Figure 1B,C), then they had disappeared 42 h after the irradiation (Figure 1D) as reported previously [5]. The distribution of l-plastin mRNA was identical to that of the AO-positive apoptotic neurons 5–24 h after the irradiation (Figure 1E–G). In a histological section of the WISH-processed brain prepared at the dotted lines shown in Figure 1G, l-plastin expressing microglia with the phagocytoic morphology (arrows in Figure 1R; arrows in Figure S1C,E) distributed in the marginal area of the irradiated OT (arrows in Figure 1R) in the same manner as that of cleaved-caspase 3 positive apoptotic neurons (arrows in Figure 1Q) and no microglia expressed l-plastin 42 h after the irradiation (Figure 1H). By contrast, no ApoE-expressing microglia were present 12 h after the irradiation (Figure 1I), and they started to appear 24 h after the irradiation (arrow in Figure 1J). Microglia expressing ApoE increased and distributed over the whole brain 42–48 h after the irradiation (Figure 1K,L). In a histological section of the WISH-processed brain of Figure 1L, the activated microglia changed their cell appearance to the migrating morphology during 24–48 h after the irradiation (arrows in Figure 1S; arrows in Figure S1D,F). Then, the ApoE-expressing microglia decreased 54 h after the irradiation (arrows in Figure 1M,N) and completely disappeared within the brain, but accumulated at the dorsal surface of the brain 60 h after the irradiation (Figure 1O,P) and maintained a rounded “amoeboid” morphology at the dorsal surface even 72 h after the irradiation (Figure S1G,H).

### 2.2. Collimated Carbon Ion Microbeam-Induced Neural Apoptosis only in the Targeted Area of OT in Medaka Embryos

**Figure 2:**
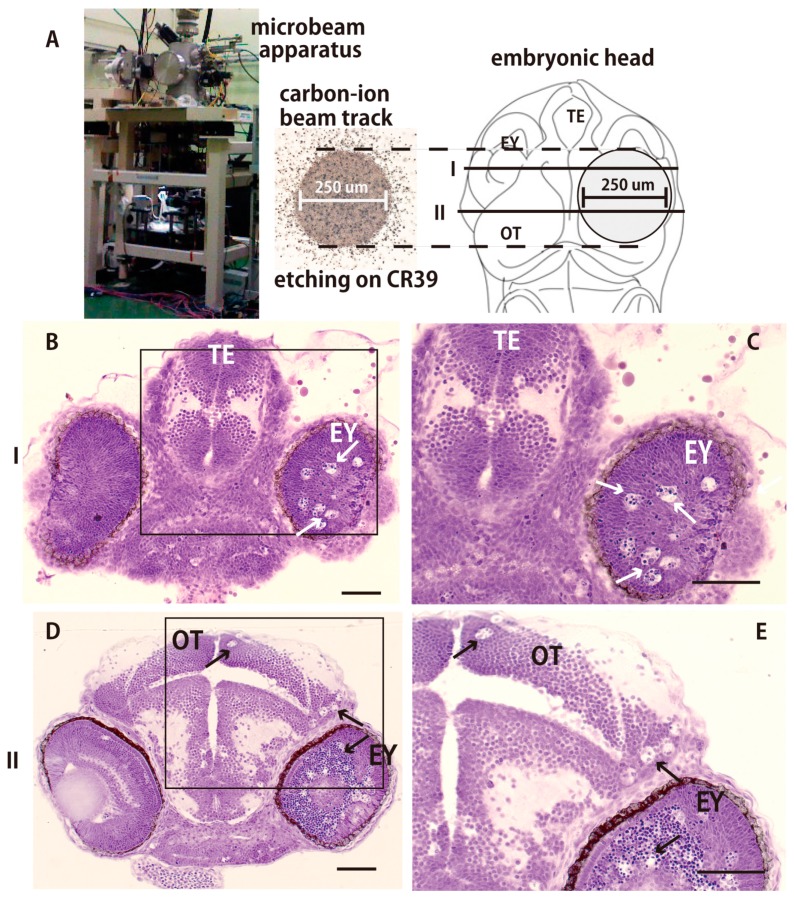
Apoptotic neuronal deaths were induced only in the targeted irradiated area 24 h after irradiation. (**A**) A collimated carbon ion microbeam (250 μm diameter) characterized by etching on CR39, was used to locally irradiate the right hemisphere of optic tectum (OT). Nissl-stained frontal sections of the locally irradiated brain were prepared at the solid lines (I and II in **A**) and shown in **B**–**E**, respectively. Many pyknotic cells were induced only in the right retina (arrows in **B**–**E**) and the right hemisphere of the OT (arrows in **D**,**E**). (**B**,**C**) show the frontal sections prepared at the solid line labeled I in **A**, and (**D**,**E**) show the frontal sections prepared at the solid line labeled II in **A**. OT, optic tectum; TE, telencephalon; EY, eye. Scale bars = 50 μm.

A collimated carbon ion microbeam (250 µm diameter), characterized by etching on CR39, was used to irradiate locally the right hemisphere of the OT of embryonic brain using the facility in TIARA of QST as shown in Figure 2A. Nissl-stained histological sections of the irradiated brain 24 h after the irradiation showed that a large number of pyknotic cells were induced only in the right retina (arrows in Figure 2B–E) and in the right hemisphere of the OT (arrows in Figure 2D,E), whereas no pyknotic cells were observed in the left hemisphere of the irradiated OT which was not irradiated.

**Figure 3:**
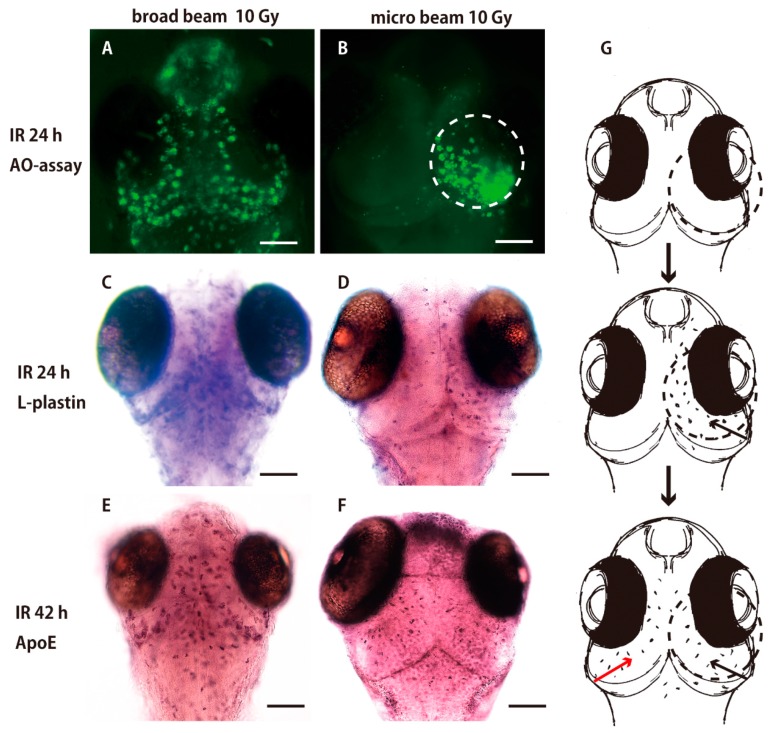
Irradiation targeted to the right hemisphere of OT of medaka embryonic brain. AO-staining of the irradiated brain 24 h after the irradiation demonstrated that AO-positive apoptotic neurons were induced only in the irradiated right hemisphere (dotted circle in **B**). By contrast, AO-positive apoptotic cells were induced throughout the whole brain when the whole embryonic brain was irradiated with broad beam (10 Gy) (**A**). When AO-positive apoptotic neurons were induced only in the irradiated right hemisphere (**B**), only the microglia positioned in the irradiated right hemisphere express l-plastin as shown by WISH (**D**), in contrast to that microglia were activated to express l-plastin in the whole brain after whole-brain irradiation (**C**). At the later phase of phagocytosis, 42 h after the irradiation, a large number of microglia express ApoE, not only in the irradiated right hemisphere (black arrows in **G**), but also in the left hemisphere (red arrow in **G**), which was not irradiated (**F**), in a similar manner to that observed after whole-brain irradiation (**E**). Schematic drawings of the abscopal effect in microglial activation are shown in **G**. Scale bars = 100 μm.

AO-staining assay of the irradiated brain 24 h after the irradiation demonstrated that the AO-positive apoptotic neurons were induced only in the irradiated right hemisphere (dotted circle in Figure 3B). By contrast, the AO-positive apoptotic cells were induced throughout the whole brain when the whole embryonic brain was irradiated with a broad beam of carbon ions (Figure 3A). This result clearly demonstrates that only the directly irradiated neurons were damaged and underwent apoptotic neural death, and that irradiation-induced apoptotic neural death was limited to the directly irradiated area.

### 2.3. Microglia in the Nonirradiated Area of OT Were Activated to Express ApoE after Irradiation

**Figure 4:**
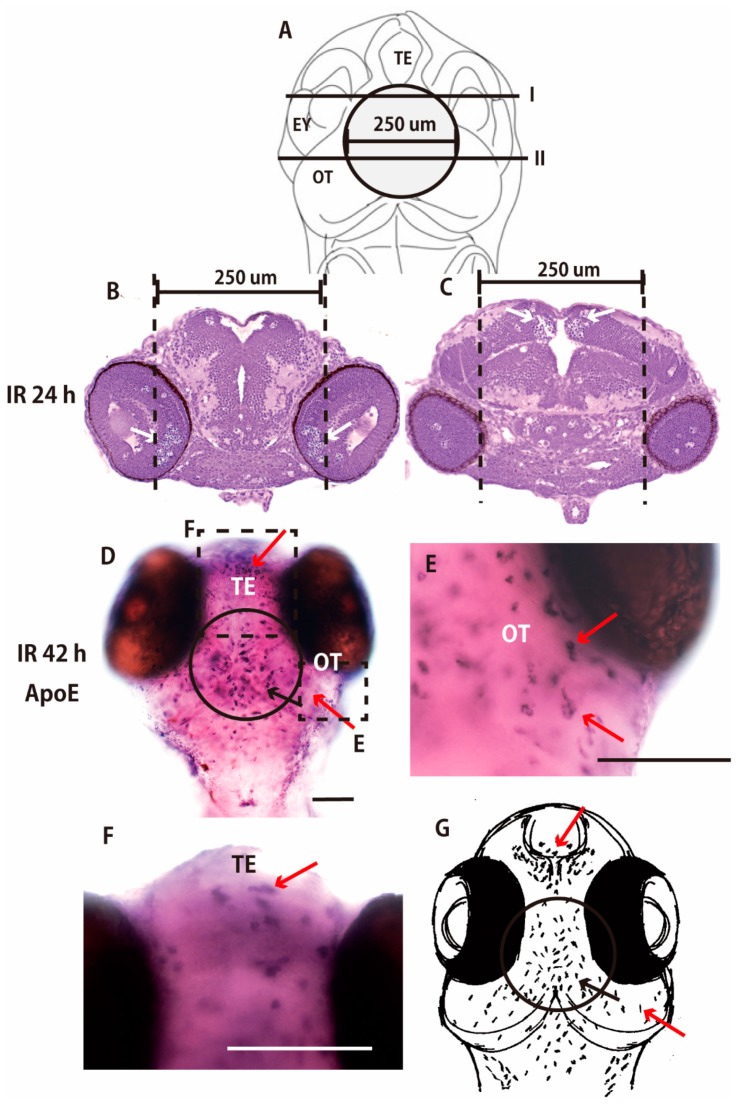
Irradiation targeted to the center of OT of medaka embryonic brain. Nissl-stained frontal sections of the brain 24 h after the targeted irradiation were prepared at the solid lines of I and II in (**A**) and shown in (**B**,**C**), respectively. Many pyknotic cells were induced in the limited area of the retina (arrows in **B**) and the central part of OT (arrows in **C**) where microbeam irradiation was targeted (dotted lines in **B**,**C**). ApoE-expressing activated microglia at the late phase of phagocytosis were present 42 h after the irradiation, not only in the irradiated area of the central part of OT (circled area in **D**), but also beyond the irradiated area of OT (red arrows in **E**) and in the telencephalon (red arrow in **F**). A schematic drawing of the abscopal effect of microglial activation is shown in **G** (red arrows in **G**). Scale bars = 100 μm.

When AO-positive apoptotic neurons were induced only in the irradiated right hemisphere 24 h after the targeted irradiation (Figure 3B), only the microglia positioned in the irradiated right hemisphere expressed l-plastin (Figure 3D), microglia were activated to express l-plastin in the whole brain 24 h after the irradiation (Figure 3C) when apoptosis was induced in the whole brain by whole-brain irradiation with broad beam carbon ions (Figure 3A). Taken together, these results suggest that the initial activation of microglia depends on the apoptotic neurons that are induced by direct damage to the irradiated neurons. Unexpectedly, at the later phase of phagocytosis, 42 h after the irradiation, we found that a large number of microglia were present expressing ApoE in the left hemisphere, which was not irradiated (Figure 4F), in a similar manner to that observed after whole-brain irradiation (Figure 4E).

To confirm the distribution of activated microglia beyond the irradiated area at the late phase of phagocytosis, we further conducted targeted irradiation to another area of the OT, directed to the central part of the OT. Nissl-stained histological sections of irradiated embryo 24 h after the irradiation demonstrated that a large number of pyknotic cells were induced in the limited area of the retina (arrows in Figure 4B) and in the central part of the OT (arrows in Figure 4C) those located in the targeted-irradiated area in the embryonic brain (Figure 4A), whereas no apoptotic neurons were observed outside the irradiated area. ApoE-expressing activated microglia at the late phase of phagocytosis, corresponding to 42 h after the irradiation, were present not only in the irradiated area of the central part of the OT (circled area in Figure 4D), but also outside of the irradiated area of OT (red arrow in Figure 4E) and in the telencephalon (red arrows in Figure 4F). These results suggest that the targeted irradiation of embryonic brain induced extensive activation of microglia at the late phase of phagocytosis as shown in the schematics in Figure 3G and Figure 4G.

## 3. Discussion

**Figure 5:**
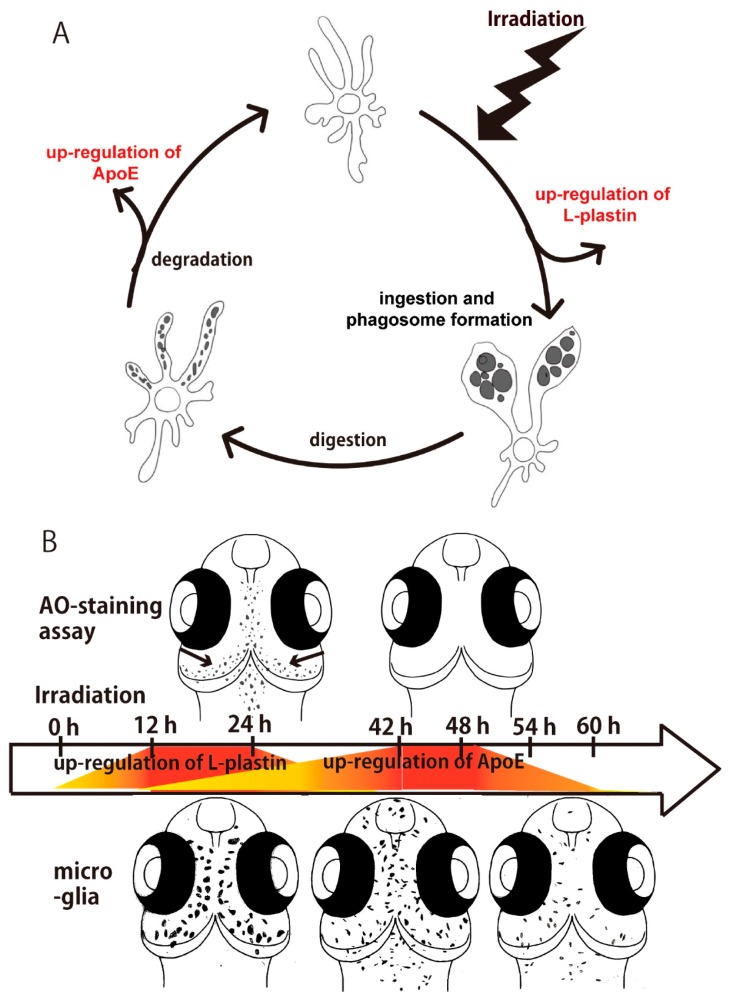
Sequential process of microglial activation in the irradiated medaka embryonic brain. (**A**) Activated microglia ingest apoptotic cells and form phagosomes to digest the cell debris. During the process of phagocytosis, l-plastin may be upregulated at the initial phase of microglial activation, especially in the process of ingestion of apoptotic cells. ApoE is expressed at the later phase of phagocytosis for clearance of cell debris in the microglial phagosomes; (**B**) Schematic images of sequential distribution of activated microglia expressing l-plastin and ApoE after irradiation.

In the present study, we investigated the microglial activation induced after γ-ray irradiation of the medaka embryonic brain and found that activated microglia express l-plastin for 5–24 h after the irradiation when microglia change their morphology from ramified to amoeboid and migrate toward the apoptotic cells. During this phase, microglia begin a process of phagocytosis of apoptotic neurons: acridine orange-stained apoptotic cells appear 3 h after the irradiation; these apoptotic cells are phagocytosed by activated microglia, which accumulate to form the rosette-shaped clusters of nuclear fragments 8–10 h after the irradiation [5,47]. By contrast, ApoE expression is upregulated in the later phase of phagocytosis (24–54 h after the irradiation) when apoptotic bodies in the phagosomes are digested and degraded [5].

l-plastin is a member of a family of actin-binding proteins and known to be an important component in cellular processes critical for immunity, such as antigen receptor signaling, adhesion, and motility in macrophage, T-lymphocyte, and the other immune cells [51]. l-plastin was upregulated when microglia migrated to the damaged neuronal area, and phagocytosed and ingested them in their phagosome. In addition, ApoE is expressed in the late phase of phagocytosis and functions in the clearance of lipids and cholesterols that are released from digested cell debris in the phagosomes of microglia [5,52]. l-plastin and ApoE have been used as molecular markers to label microglia in zebrafish [9,28,53], and the present results indicate that l-plastin and ApoE may be biomarkers of activated microglia in the early and late phases of phagocytosis, respectively as illustrated in Figure 5A. Here, we demonstrated sequential process of microglial activation induced after γ-ray irradiation with 10 Gy by using these two biomarkers as illustrated in Figure 5B. Medaka represent a unique model system with which to investigate the molecular and cellular mechanism of microglial activation in detail.

The collimated carbon ion microbeam irradiation system at the facility of TIARA, QST enables us to locally irradiate a very small region of tissue or even a single cell [54], which is useful for research on radiation biology [55]. In the present study, using this system, we locally irradiated the right half-hemisphere of the OT of the medaka embryonic brain. As shown by AO staining and histological sections, the local irradiation using the collimated microbeam system reliably and reproducibly induced apoptotic cell death in the region restricted to that which was irradiated, so we could conduct targeted irradiation of the embryonic medaka brain to reveal dynamics of microglia in response to irradiation.

We found that l-plastin-expressing microglia localized in the right hemisphere of OT, which was irradiated and where damaged neuronal cells were induced, for 12–24 h after the irradiation. Thereafter, we also found that 24–48 h after the irradiation, ApoE-expressing microglia were present throughout the whole brain, including in the left hemisphere of OT, which was not irradiated. Since it is widely accepted that microglia are activated by diffusible molecular signals from apoptotic cells [11,21,22], it can be assumed that the upregulation of l-plastin and ApoE are the sequential steps of microglial activation and that the activated microglia migrated out to the left hemisphere of the OT in the late phase of phagocytosis expressing ApoE. In our previous study in the p53-deficient irradiated embryonic brain [5], as many microglia as in wild-type embryos migrated through the whole brain after the irradiation expressing ApoE, even though very few apoptotic cells were induced after γ-ray irradiation in p53-deficient embryos. This finding strongly suggests that microglial activation includes regenerative processes: a microglial cell which was activated in response to apoptotic cell death activates the resting microglia around it, and the microglial activation is amplified to cause activation of all microglia. Taken together with the findings obtained in this study, this suggests that in the locally irradiated right hemisphere of the OT, and in the locally irradiated center of OT, the irradiation-induced apoptotic cell death might activate microglia located near apoptotic cells, and that these microglia then migrate out to the whole brain, further activating resting microglia outside of the irradiated area.

In the late phase of phagocytosis, the expression of ApoE in microglia was downregulated 54 h after the irradiation and ApoE-expressing microglia disappeared from the brain by 60 h after the irradiation. The recruitment of activated microglia was sustained for longer than 10 h even after injured neurons had been largely eliminated at 42 h after irradiation. As shown in Figure 1O,P in this paper, ApoE-expressing microglia accumulated in the dorsal surface of the brain and their morphology were not ramified, but amoeboid, indicating they were still activated even 72 h after the irradiation. Previous studies *in vivo* in mice have found similar results. Irradiation induced a long-term increase in microglial recruitment to the irradiated area, by contrast with a transient increase induced when the brain was injured locally [56]. Stroke is a common age-related disease in human and the suitable animal models for stroke have been established [57,58]. In aged rat brain, it was shown that upregulation of phagocytosis-specific proteins, such as annexin A1 and A3, are induced in the infarct area after stroke or ischemia, indicating that the phagocytotic activity by not microglia but polymorphonuclear cells is activated primarily after stroke or ischemia in aged rat brain [59,60]. On the other hand, recent experimental evidence suggests that the microenvironment of the normal aged brain is characterized by chronic low-level inflammation and increased microglia reactivity [57], which may exacerbate post-stroke inflammation by creating a primed inflammatory environment in the brain [61]. In aged rat brain, stroke induced the delayed activation of microglia in the peri-infarcted area [58]. Taken together with the present finding that microglia can be “primed” for days after the irradiation-induced neural injury, it is very possible that microglia significantly contribute in the cellular events induced against brain injury caused by stroke, ischemia and irradiation.

Radiotherapy is generally administrated for cancer treatment by localized targeted irradiation [36,39]. The results reported here show that targeted irradiation induced abscopal activation of the immune system in the embryonic brains of medaka. Another study in mice demonstrated that the persistent microglial activation, even 6 weeks after irradiation, plays an important role in RIBI, inducing the upregulation of the proinflammatory genes. Furthermore, the authors demonstrated that inhibition of microglial activation can reduce radiation-induced detrimental effects, such as cognitive deficits [62]. Our present results showing the abscopal activation of the immune system in the embryonic brain after irradiation suggest that chemical signals such as proinflammatory cytokines might be released from the activated microglia in the irradiated area affecting microglia in the nonirradiated area. The embryonic medaka brain can provide a unique vertebrate model system *in vivo* with which to investigate cranial immune responses following targeted irradiation and the cellular machinery of the abscopal or adverse effects of irradiation. A better understanding of the mechanisms driving the interactions between IR and the immune system would be very helpful to develop more powerful strategies for cancer treatment.

## 4. Materials and Methods

### 4.1. Ethics

This research was conducted using protocols approved by the Animal Care and Use Committee of the University of Tokyo (permit number: C-09-01, 19 May 2009). All surgery on embryos was performed using chilling as anesthesia, and all efforts were made to minimize suffering.

### 4.2. Fish and Embryos

An Hd-rR inbred strain of medaka, established from a southern Japanese population [63], was kept in our laboratory. The fish were maintained at 26–28 °C under a 14 h light and 10 h dark cycle, and fed on a powdered diet (TetraFin, Spectrum Brands Japan, Yokohama, Japan) and brine shrimp (*Artemia franciscana*) three times per day.

Female medaka spawn eggs every morning. Egg clusters were collected and rubbed between two small pieces of paper towel to remove filaments on the chorion; the isolated eggs were then incubated in a petri dish filled with 7 mL of distilled water containing 10^−5^% (*w*/*v*) methylene blue at 26–28 °C. The developmental stages of the embryos are described according to Iwamatsu [64].

### 4.3. Irradiation

Embryos at stage 28 (30-somite stage, 64 h after fertilization) were irradiated with γ-ray emitted by ^137^Cs (10 Gy, Gammacell 3000Elan, MDS Nordion, Ottawa, ON, Canada) at a dose rate of 7.5 Gy/min at room temperature in a plastic tube containing water. Medaka embryos at stage 28 correspond approximately to early fetal stage human embryos (approximately 8–15 weeks postovulation) [65]. Targeted irradiation to the right hemisphere of OT and the central part of OT of embryonic brain with a collimated microbeam (250 μm diameter) of carbon ions was conducted at the irradiation facility of TIARA (Takasaki Ion Accelerators for Advanced Radiation Application), National Institutes of Quantum Beam Science and Technology (QST) as previously reported [54]. Irradiation of whole brain of medaka embryo was conducted as reported by Nagata et al. [44].

### 4.4. Acridine Orange Staining Assay

Acridine orange (AO) (Sigma-Aldrich, St Louis, MO, USA), a single-strand DNA intercalating vital dye, selectively stains the nuclei of apoptotic cells, but does not significantly label those of necrotic cells [66,67]. The irradiated embryos were stained with AO (17 μg/mL) as described previously [48,68]. The AO-stained embryos were observed using a fluorescence microscope (BX50, Olympus, Tokyo, Japan) with an appropriate filter (U-MGFPHQ, Olympus), 460–480 nm excitation, and 495–540 nm emission wavelengths, equipped with a digital still camera (DP70, Olympus, Tokyo, Japan).

### 4.5. Histology

Medaka embryos were anesthetized by chilling and fixed in 4% (*w*/*v*) paraformaldehyde in 0.1 M phosphate buffer overnight at 0–4 °C. The fixed embryos were dehydrated in an ethanol series, embedded in plastic resin (Technovit 8100, Heraeus Kulzer, Wehrheim, Germany), and sectioned frontally into a complete series of 8 μm serial sections as described previously [47,69]. The sections were Nissl-stained with cresyl violet for light microscopy.

### 4.6. Immunohistochemistry

The embryo and adult brains used for immunohistochemistry were prepared as previously reported [47]. Serial sections (20 μm thickness) were cut on a cryostat, nonspecific binding sites were blocked by incubation in phosphate-buffered saline (PBS) containing normal goat serum for 30 min at room temperature, and the sections were then washed in PBS and incubated with a polyclonal anti-cleaved caspase-3 antibody (9661S, Cell Signaling Technology, Danvers, MA, USA) (1:200) for 3 h at room temperature. The sections were further incubated with secondary antibodies conjugated to Alexa-488 (A11001, Invitrogen, Carlsbad, CA, USA) for fluorescent immunohistochemistry and counterstained with 4,6-diamidino-2-phenylindole (DAPI). Images were obtained using a fluorescence microscope (BX50, Olympus, Tokyo, Japan), equipped with a digital camera (DFC7000T, Leica, Wetzlar, Germany).

### 4.7. Whole-Mount In Situ Hybridization (WISH)

The sequences of the medaka ApoE and l-plastin genes were obtained from the Ensembl Genome Browser database (http://asia.ensembl.org/index.html). A DNA fragment of these genes was amplified using polymerase chain reaction (PCR) from Hd-rR cDNA, with primer pairs as follows: ApoE forward, 5’–CGAAACCATGACTGAGGTGA–3’ and reverse, 5’–AACCCTCAAAAACCCCAAGT–3’; l-plastin forward, 5’–ACCTTCAGGAAAGCCATCAA–3’ and reverse, 5’–ATTCACACCTAGAGAGTTCATCCA–3’. The PCR reactions were performed for 30 cycles at 95 °C for 30 s, 60 °C (ApoE) or 55 °C (l-plastin) for 30 s, and 72 °C for 1 min. The amplified ApoE and l-plastin cDNA sequences were cloned into pCR4-TOPO, digested with *Not*I, and transcribed in vitro with T3 polymerase to prepare DIG-labeled RNA probes for WISH. Embryos stained with the l-plastin and ApoE probe 24 and 48 h after the irradiation were embedded in Technovit 8100, serially sectioned (at 8 μm thickness), and counterstained with 0.5% neutral red (Muto Pure Chemicals, Tokyo, Japan) before microscopy as previously reported [5].

## 5. Conclusions

Here, we established the experimental procedures to locally irradiate the embryonic brain of OT locally, using the collimated carbon-ion beam system at the facility of TIARA in National Institutes of QST, and investigated the dynamics of microglial activities induced after the target irradiation. We demonstrated that the microglial activation includes two steps throughout the phagocytotic process, which can be indicated by the specific two biomarkers, l-plastin and ApoE. Furthermore, microglial activation was induced, not only in the target irradiation area of OT, but also in the non-irradiated area of OT. These findings strongly suggest that the abscopal activation of brain immune system could be induced after cranial radiation therapy in humans.

## Supplementary Materials

Supplementary materials can be found at www.mdpi.com/1422-0067/18/7/1428/s1.

## References

[1] Ransohoff R.M., Cardona A.E. (2010). The myeloid cells of the central nervous system parenchyma. Nature.

[2] Eyo U., Dailey M.E. (2012). Effects of oxygen-glucose deprivation on microglial mobility and viability in developing mouse hippocampal tissues. Glia.

[3] Prinz M., Priller J. (2014). Microglia and brain macrophages in the molecular age: From origin to neuropsychiatric disease. Nat. Rev. Neurosci..

[4] Lyons D.A., Talbot W.S. (2014). Glial cell development and function in zebrafish. Cold Spring Harb. Perspect. Biol..

[5] Yasuda T., Oda S., Hibi Y., Satoh S., Nagata K., Hirakawa K., Kutsuna N., Sagara H., Mitani H. (2015). Embryonic medaka model of microglia in the developing cns allowing in vivo analysis of their spatiotemporal recruitment in response to irradiation. PLoS ONE.

[6] Morrens J., Van Den Broeck W., Kempermann G. (2012). Glial cells in adult neurogenesis. Glia.

[7] Huang T., Cui J.L., Li L., Hitchcock P.F., Li Y.H. (2012). The role of microglia in the neurogenesis of zebrafish retina. Biochem. Biophys. Res. Commun..

[8] Sieger D., Peri F. (2013). Animal models for studying microglia: The first, the popular, and the new. Glia.

[9] Peri F., Nusslein-Volhard C. (2008). Live imaging of neuronal degradation by microglia reveals a role for v0-ATPase a1 in phagosomal fusion in vivo. Cell.

[10] Ellett F., Pase L., Hayman J.W., Andrianopoulos A., Lieschke G.J. (2011). mpeg1 promoter transgenes direct macrophage-lineage expression in zebrafish. Blood.

[11] Davalos D., Grutzendler J., Yang G., Kim J.V., Zuo Y., Jung S., Littman D.R., Dustin M.L., Gan W.B. (2005). ATP mediates rapid microglial response to local brain injury in vivo. Nat. Neurosci..

[12] Nimmerjahn A., Kirchhoff F., Helmchen F. (2005). Resting microglial cells are highly dynamic surveillants of brain parenchyma in vivo. Science.

[13] Li Y., Du X.F., Liu C.S., Wen Z.L., Du J.L. (2012). Reciprocal regulation between resting microglial dynamics and neuronal activity in vivo. Dev. Cell.

[14] Hanisch U.K., Kettenmann H. (2007). Microglia: Active sensor and versatile effector cells in the normal and pathologic brain. Nat. Neurosci..

[15] Marz M., Schmidt R., Rastegar S., Strahle U. (2011). Regenerative response following stab injury in the adult zebrafish telencephalon. Dev. Dyn..

[16] Baumgart E.V., Barbosa J.S., Bally-Cuif L., Gotz M., Ninkovic J. (2012). Stab wound injury of the zebrafish telencephalon: A model for comparative analysis of reactive gliosis. Glia.

[17] Oosterhof N., Holtman I.R., Kuil L.E., van der Linde H.C., Boddeke E.W., Eggen B.J., van Ham T.J. (2017). Identification of a conserved and acute neurodegeneration-specific microglial transcriptome in the zebrafish. Glia.

[18] Sieger D., Moritz C., Ziegenhals T., Prykhozhij S., Peri F. (2012). Long-range Ca^2+^ waves transmit brain-damage signals to microglia. Dev. Cell.

[19] Hamilton L., Astell K.R., Velikova G., Sieger D. (2016). A zebrafish live imaging model reveals differential responses of microglia toward glioblastoma cells in vivo. Zebrafish.

[20] Astell K.R., Sieger D. (2017). Investigating microglia-brain tumor cell interactions in vivo in the larval zebrafish brain. Methods Cell Biol..

[21] Haynes S.E., Hollopeter G., Yang G., Kurpius D., Dailey M.E., Gan W.B., Julius D. (2006). The P2Y12 receptor regulates microglial activation by extracellular nucleotides. Nat. Neurosci..

[22] Elliott M.R., Chekeni F.B., Trampont P.C., Lazarowski E.R., Kadl A., Walk S.F., Park D., Woodson R.I., Ostankovich M., Sharma P. (2009). Nucleotides released by apoptotic cells act as a find-me signal to promote phagocytic clearance. Nature.

[23] Davis E.J., Foster T.D., Thomas W.E. (1994). Cellular forms and functions of brain microglia. Brain Res. Bull..

[24] Svahn A.J., Graeber M.B., Ellett F., Lieschke G.J., Rinkwitz S., Bennett M.R., Becker T.S. (2013). Development of ramified microglia from early macrophages in the zebrafish optic tectum. Dev. Neurobiol..

[25] Li Y., Du X., Pei G., Du J., Zhao J. (2016). β-Arrestin1 regulates the morphology and dynamics of microglia in zebrafish in vivo. Eur. J. Neurosci..

[26] Cai Q., Li Y., Mao J., Pei G. (2016). Neurogenesis-promoting natural product α-Asarone modulates morphological dynamics of activated microglia. Front. Cell. Neurosci..

[27] Mazaheri F., Breus O., Durdu S., Haas P., Wittbrodt J., Gilmour D., Peri F. (2014). Distinct roles for BAI1 and TIM-4 in the engulfment of dying neurons by microglia. Nat. Commun..

[28] Van Ham T.J., Brady C.A., Kalicharan R.D., Oosterhof N., Kuipers J., Veenstra-Algra A., Sjollema K.A., Peterson R.T., Kampinga H.H., Giepmans B.N. (2014). Intravital correlated microscopy reveals differential macrophage and microglial dynamics during resolution of neuroinflammation. Dis. Model. Mech..

[29] Morsch M., Radford R., Lee A., Don E.K., Badrock A.P., Hall T.E., Cole N.J., Chung R. (2015). In vivo characterization of microglial engulfment of dying neurons in the zebrafish spinal cord. Front. Cell. Neurosci..

[30] Welzel G., Fleckenstein K., Mai S.K., Hermann B., Kraus-Tiefenbacher U., Wenz F. (2008). Acute neurocognitive impairment during cranial radiation therapy in patients with intracranial tumors. Strahlenther. Onkol..

[31] Greene-Schloesser D., Moore E., Robbins M.E. (2013). Molecular pathways: Radiation-induced cognitive impairment. Clin. Cancer Res..

[32] Makale M.T., McDonald C.R., Hattangadi-Gluth J.A., Kesari S. (2017). Mechanisms of radiotherapy-associated cognitive disability in patients with brain tumours. Nat. Rev. Neurol..

[33] Liu J.L., Tian D.S., Li Z.W., Qu W.S., Zhan Y., Xie M.J., Yu Z.Y., Wang W., Wu G. (2010). Tamoxifen alleviates irradiation-induced brain injury by attenuating microglial inflammatory response in vitro and in vivo. Brain Res..

[34] Xue J., Dong J.H., Huang G.D., Qu X.F., Wu G., Dong X.R. (2014). NF-κB signaling modulates radiationinduced microglial activation. Oncol. Rep..

[35] Zhang J., Tong F., Cai Q., Chen L.J., Dong J.H., Wu G., Dong X.R. (2015). Shenqi Fuzheng Injection attenuates irradiation-induced brain injury in mice via inhibition of the NF-κB signaling pathway and microglial activation. Acta Pharmacol. Sin..

[36] Formenti S.C., Demaria S. (2009). Systemic effects of local radiotherapy. Lancet Oncol..

[37] Postow M.A., Callahan M.K., Barker C.A., Yamada Y., Yuan J., Kitano S., Mu Z., Rasalan T., Adamow M., Ritter E. (2012). Immunologic correlates of the abscopal effect in a patient with melanoma. N. Engl. J. Med..

[38] Durante M., Reppingen N., Held K.D. (2013). Immunologically augmented cancer treatment using modern radiotherapy. Trends Mol. Med..

[39] Formenti S.C., Demaria S. (2013). Combining radiotherapy and cancer immunotherapy: A paradigm shift. J. Natl. Cancer Inst..

[40] Bernier J. (2016). Immuno-oncology: Allying forces of radio- and immuno-therapy to enhance cancer cell killing. Crit. Rev. Oncol. Hematol..

[41] McGinnis G.J., Friedman D., Young K.H., Torres E.R., Thomas C.R., Gough M.J., Raber J. (2017). Neuroinflammatory and cognitive consequences of combined radiation and immunotherapy in a novel preclinical model. Oncotarget.

[42] Wittbrodt J., Shima A., Schartl M. (2002). Medaka-a model organism from the far East. Nat. Rev. Genet..

[43] Suzuki N., Nawa D., Tateno H., Yasuda T., Oda S., Mitani H., Nishimaki T., Katsumura T., Oota H., Hanihara T. (2013). Generation of monoclonal antibodies against the Galβ1–4Gal epitope: A key tool in studies of species-specific glycans expressed in fish, amphibians and birds. Glycobiology.

[44] Nagata K., Hashimoto C., Watanabe-Asaka T., Itoh K., Yasuda T., Ohta K., Oonishi H., Igarashi K., Suzuki M., Funayama T. (2016). In vivo 3D analysis of systemic effects after local heavy-ion beam irradiation in an animal model. Sci. Rep..

[45] Ishikawa Y. (2000). Medakafish as a model system for vertebrate developmental genetics. Bioessays.

[46] Shima A., Mitani H. (2004). Medaka as a research organism: Past, present and future. Mech. Dev..

[47] Yasuda T., Oda S., Ishikawa Y., Watanabe-Asaka T., Hidaka M., Yasuda H., Anzai K., Mitani H. (2009). Live imaging of radiation-induced apoptosis by yolk injection of Acridine orange in the developing optic tectum of medaka. J. Radiat. Res..

[48] Yasuda T., Oda S., Yasuda H., Hibi Y., Anzai K., Mitani H. (2011). Neurocytotoxic effects of iron-ions on the developing brain measured in vivo using medaka (*Oryzias latipes*), a vertebrate model. Int. J. Radiat. Biol..

[49] Yasuda T., Oda S., Li Z., Kimori Y., Kamei Y., Ishikawa T., Todo T., Mitani H. (2012). γ-ray irradiation promotes premature meiosis of spontaneously differentiating testis-ova in the testis of p53-deficient medaka (*Oryzias latipes*). Cell Death Dis..

[50] Zheng H., Liu R., Zhang R., Hu Y. (2014). A method for real-time measurement of respiratory rhythms in medaka (*Oryzias latipes*) using computer vision for water quality monitoring. Ecotoxicol. Environ. Saf..

[51] Morley S.C. (2013). The actin-bundling protein l-plastin supports T-cell motility and activation. Immunol. Rev..

[52] Leblanc A.C., Poduslo J.F. (1990). Regulation of apolipoprotein-E gene-expression after injury of the rat sciatic-nerve. J. Neurosci. Res..

[53] Herbomel P., Thisse B., Thisse C. (2001). Zebrafish early macrophages colonize cephalic mesenchyme and developing brain, retina, and epidermis through, a M-CSF receptor-dependent invasive process. Dev. Biol..

[54] Funayama T., Wada S., Yokota Y., Fukamoto K., Sakashita T., Taguchi M., Kakizaki T., Hamada N., Suzuki M., Furusawa Y. (2008). Heavy-ion microbeam system at JAEA-Takasaki for microbeam biology. J. Radiat. Res..

[55] Furusawa T., Fukamoto K., Sakashita T., Suzuki E., Kakizaki T., Hamada N., Funayama T., Suzuki H., Ishioka N., Wada S. (2009). Targeted heavy-ion microbeam irradiation of the embryo but not yolk in the diapause-terminated egg of the silkworm, *bombyx mori*, induces the somatic mutation. J. Radiat. Res..

[56] Burrell K., Hill R.P., Zadeh G. (2012). High-resolution in vivo analysis of normal brain response to cranial irradiation. PLoS ONE.

[57] Buga A.M., Di Napoli M., Popa-Wagner A. (2013). Preclinical models of stroke in aged animals with or without comorbidities: Role of neuroinflammation. Biogerontology.

[58] Sandu R.E., Buga A.M., Balseanu A.T., Moldovan M., Popa-Wagner A. (2016). Twenty-four hours hypothermia has temporary efficacy in reducing brain infarction and inflammation in aged rats. Neurobiol. Aging.

[59] Junker H., Suofu Y., Venz S., Sascau M., Herndon J.G., Kessler C., Walther R., Popa-Wagner A. (2007). Proteomic identification of an upregulated isoform of annexin A3 in the rat brain following reversible cerebral ischemia. Glia.

[60] Joseph C., Buga A.M., Vintilescu R., Balseanu A.T., Moldovan M., Junker H., Walker L., Lotze M., Popa-Wagner A. (2012). Prolonged gaseous hypothermia prevents the upregulation of phagocytosis-specific protein Annexin 1 and causes low-amplitude EEG activity in the aged rat brain after cerebral ischemia. J. Cerebr. Blood Flow Metab..

[61] Amor S., Puentes F., Baker D., van der Valk P. (2010). Inflammation in neurodegenerative diseases. Immunology.

[62] Acharya M.M., Green K.N., Allen B.D., Najafi A.R., Syage A., Minasyan H., Le M.T., Kawashita T., Giedzinski E., Parihar V.K. (2016). Elimination of microglia improves cognitive function following cranial irradiation. Sci. Rep..

[63] Hyodo-Taguchi Y., Egami N. (1985). Establishment of inbred strains of the medaka *Oryzias latipes* and the usefulness of the strains for biomedical-research. Zool. Sci..

[64] Iwamatsu T. (2004). Stages of normal development in the medaka *Oryzias latipes*. Mech. Dev..

[65] Ishikawa Y., Yamamoto N., Yoshimoto M., Yasuda T., Maruyama K., Kage T., Takeda H., Ito H. (2007). Developmental origin of diencephalic sensory relay nuclei in teleosts. Brain Behav. Evol..

[66] Abrams J.M., White K., Fessler L.I., Steller H. (1993). Programmed cell death during Drosophila embryogenesis. Development.

[67] Furutani-Seiki M., Jiang Y.J., Brand M., Heisenberg C.P., Houart C., Beuchle D., van Eeden F.J., Granato M., Haffter P., Hammerschmidt M. (1996). Neural degeneration mutants in the zebrafish, *Danio rerio*. Development.

[68] Yasuda T., Yoshimoto M., Maeda K., Matsumoto A., Maruyama K., Ishikawa Y. (2008). Rapid and simple method for quantitative evaluation of neurocytotoxic effects of radiation on developing Medaka brain. J. Radiat. Res..

[69] Yasuda T., Aoki K., Matsumoto A., Maruyama K., Hyodo-Taguchi Y., Fushiki S., Ishikawa Y. (2006). Radiation-induced brain cell death can be observed in living Medaka embryos. J. Radiat. Res..

