# Supplementary material for: Abscopal Activation of Microglia in Embryonic Fish Brain Following Targeted Irradiation with Heavy-Ion Microbeam
Source: Int J Mol Sci. 2017 Jul 4;18(7):1428. doi: 10.3390/ijms18071428 (PMC5535919; doi:10.3390/ijms18071428)
Supplement: Supplementary file 1 [file ijms-18-01428-s001.pdf]

International Journal of
Molecular Sciences

MDPI

Abscopal Activation of Microglia in Embryonic Fish Brain following Targeted Irradiation with Heavy-Ion Microbeam

Takako Yasuda, Miyuki Kamahori, Kento Nagata, Tomomi Watanabe-Asaka, Michiyo Suzuki, Tomoo Funayama, Hiroshi Mitani and Shoji Oda

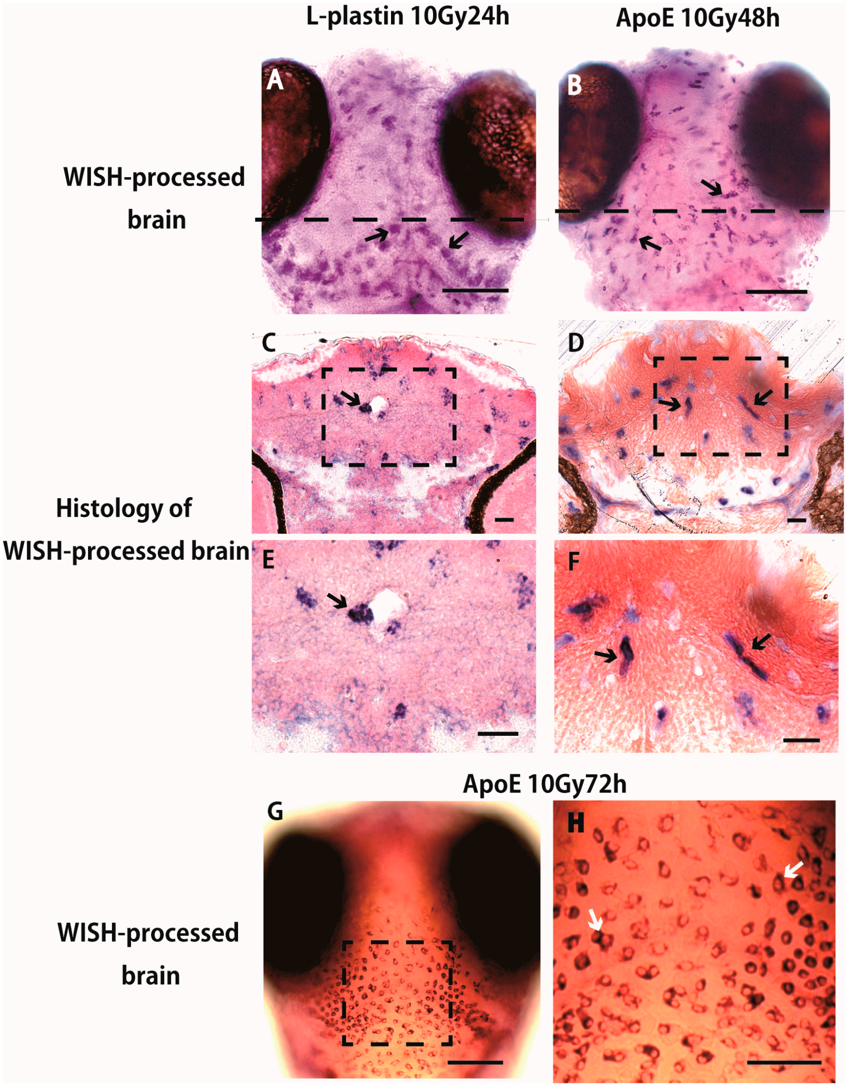

Figure S1. The morphology of microglia during 3 days after γ-ray irradiation. The distribution of L-plastin mRNA 24 h after the irradiation (**A**) and ApoE mRNA 48 h after the irradiation (**B**) were revealed by WISH. In a histological section of the WISH-processed brain prepared at the dotted line in A, L-plastin expressing microglia showed the phagocytoic morphology (arrows in **C** and **E**). A magnified image of the microglia with the phagocytoic morphology in the area squared with dotted line in C is showed in E. In a histological section of the WISH-processed brain prepared at the dotted line in B, ApoE expressing microglia showed migrating morphology (arrows in **D** and **F**). A magnified image of the microglia with the migrating morphology of microglia in the area squared with dotted line in D is showed in F. ApoE-expressing microglia accumulated at the dorsal surface of the brain and maintained a rounded "amoeboid" morphology even 72 h after the irradiation (**G**, arrows in **H**). A magnified image of the "amoeboid" microglia in the area squared with dotted line in G is showed in H. Scale bars = 100 μm in A, B and G; = 20 μm in C- F and H.
